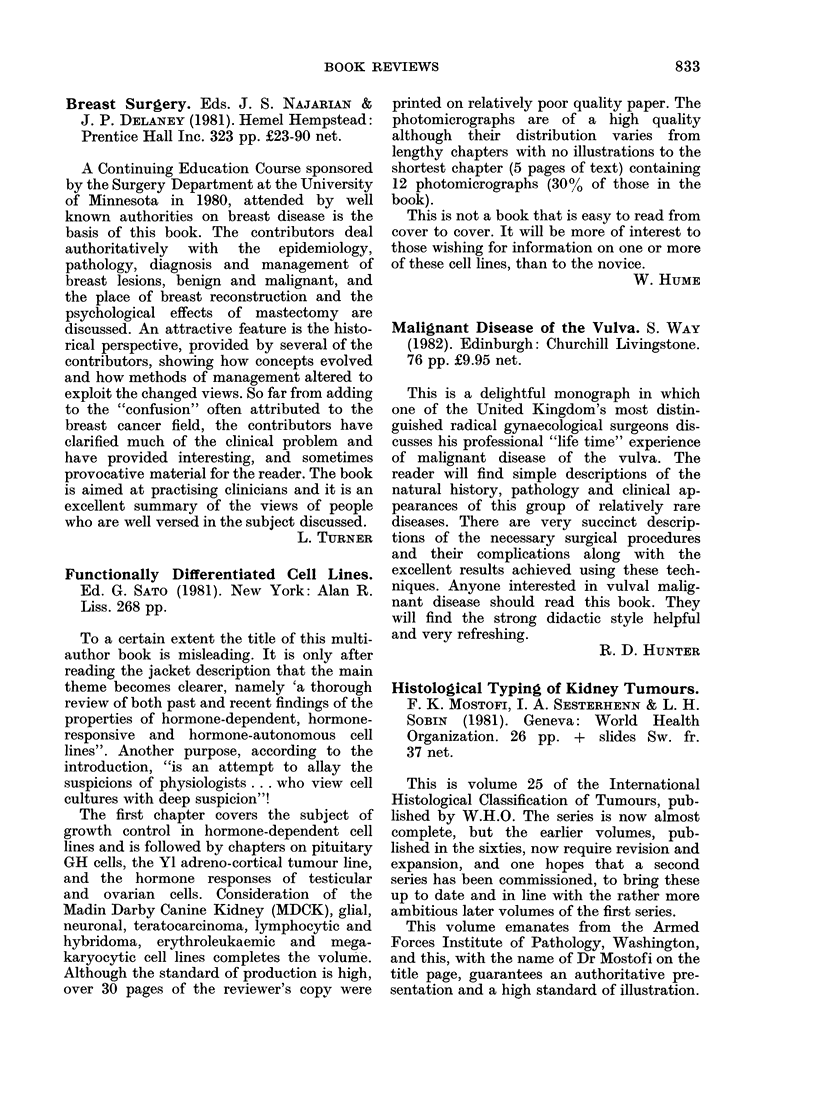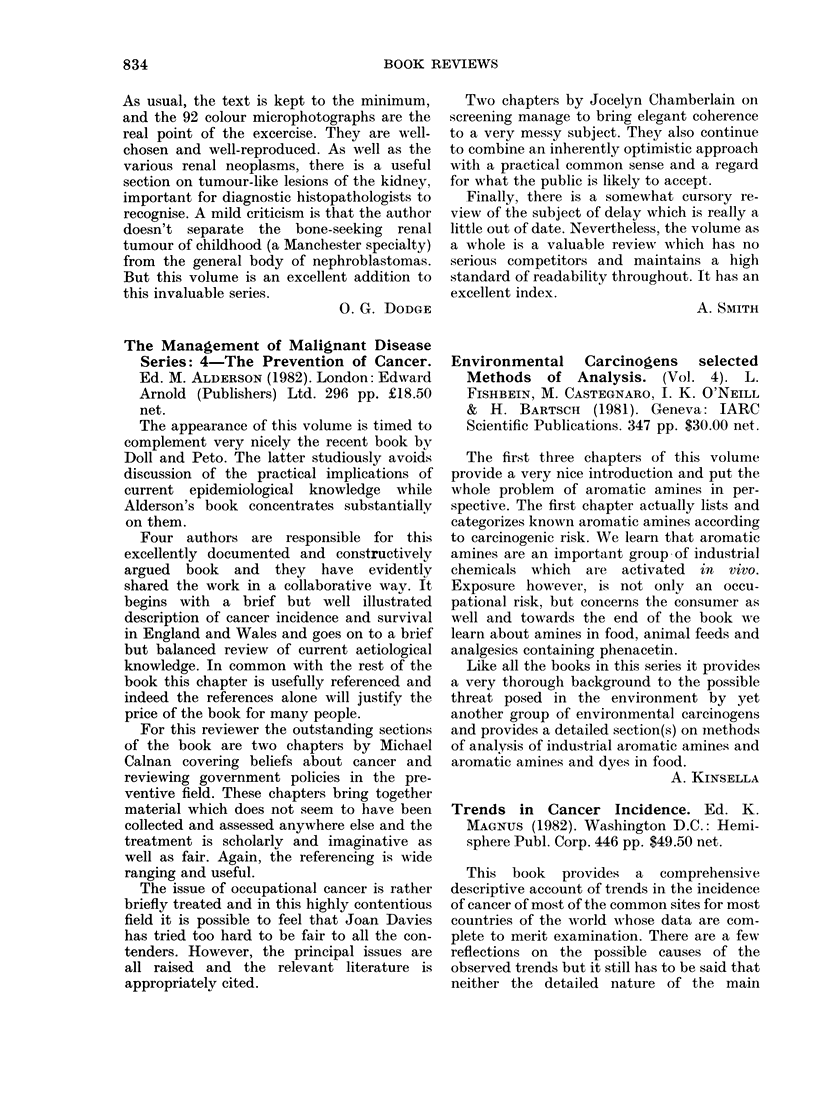# Histological Typing of Kidney Tumours

**Published:** 1982-11

**Authors:** O. G. Dodge


**Histological Typing of Kidney Tumours.** F. K. MOSTOFI, I. A. SESTERHENN & L. H. SOBIN (1981). Geneva: World Health Organization. 26 pp. + slides Sw. fr. 37 net.

This is volume 25 of the International Histological Classification of Tumours, published by W.H.O. The series is now almost complete, but the earlier volumes, published in the sixties, now require revision and expansion, and one hopes that a second series has been commissioned, to bring these up to date and in line with the rather more ambitious later volumes of the first series.

This volume emanates from the Armed Forces Institute of Pathology, Washington, and this, with the name of Dr Mostofi on the title page, guarantees an authoritative presentation and a high standard of illustration.

As usual, the text is kept to the minimum, and the 92 colour microphotographs are the real point of the excercise. They are well-chosen and well-reproduced. As well as the various renal neoplasms, there is a useful section on tumour-like lesions of the kidney, important for diagnostic histopathologists to recognise. A mild criticism is that the author doesn't separate the bone-seeking renal tumour of childhood (a Manchester specialty) from the general body of nephroblastomas. But this volume is an excellent addition to this invaluable series.

O. G. DODGE